# Supplementary material for: α-Amidoamids as New Replacements of Antibiotics—Research on the Chosen K12, R2–R4 E. coli Strains
Source: Materials (Basel). 2020 Nov 16;13(22):5169. doi: 10.3390/ma13225169 (PMC7697494; doi:10.3390/ma13225169)
Supplement: Supplementary file 1 [file materials-13-05169-s001.pdf]

materials

MDPI

Supplementary

α-Amidoamids as New Replacements of Antibiotics—Research on the Chosen K12, R2–R4 *E. coli* Strains

Paweł Kowalczyk [1,*], Arleta Madej [2], Mateusz Szymczak [3] and Ryszard Ostaszewski [2,*]

[1] Department of Animal Nutrition, The Kielanowski Institute of Animal Physiology and Nutrition, Polish Academy of Sciences, Instytucka 3, 05-110 Jabłonna, Poland
[2] Institute of Organic Chemistry, Polish Academy of Sciences, Kasprzaka 44/52, 01-224 Warsaw, Poland; arleta.madej@icho.edu.pl
[3] Department of Molecular Virology, Institute of Microbiology, Faculty of Biology, University of Warsaw, Miecznikowa 1, 02-096 Warsaw, Poland; mszymczak@biol.uw.edu.pl
* Correspondence: p.kowalczyk@ifzz.pl (P.K.); rostaszewski@icho.edu.pl (R.O.)

Below, the compounds **5a**-**5u** are numbered as 1-20, which corresponds to:
1 = **5b**, 2 = **5k**, 3 = **5a**, 4 = **5p**, 5 = **5j**, 6 = **5o**, 7 = **5g**, 8 = **5f**, 9 = **5d**, 10 = **5e**, 11 = **5c**, 12 = **5i**, 13 = **5m**, 14=**5l**, 15=**5h**, 16 = **5r**, 17 = **5s**, 18 = **5t**, 19 = **5n**, 20 = **5u**.

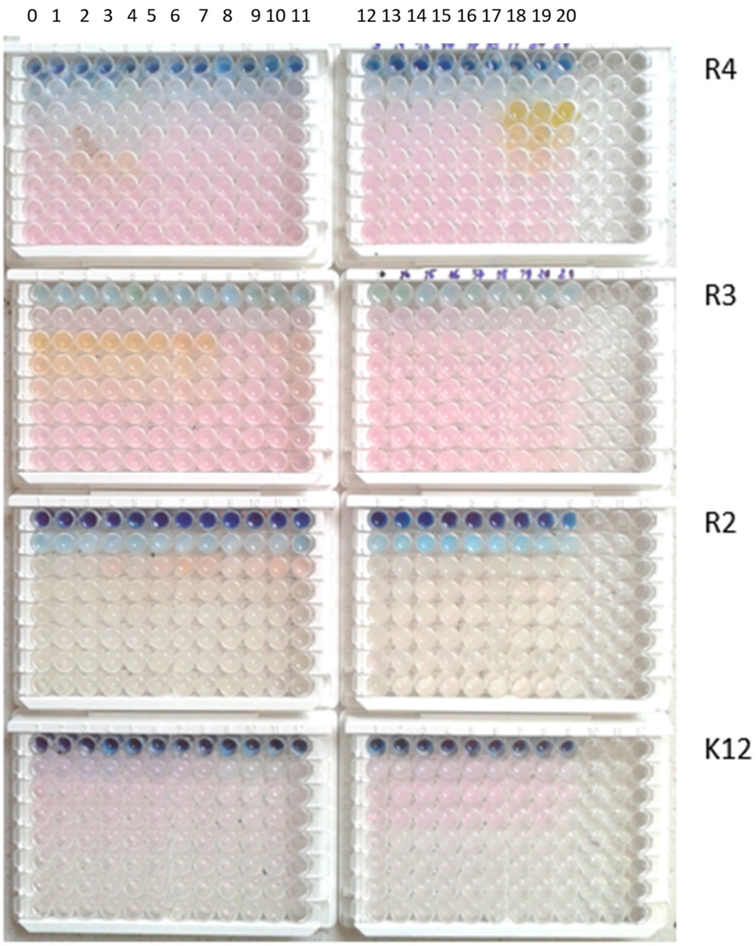

Figure S1. Examples of MIC (Minimum inhibitory concentratio). R4 strains, R2 strains, R3 strains, K12 strains with the tested first 20 compounds (wells from 1–20). Wells estimated as 0- control of compounds with reference strain.

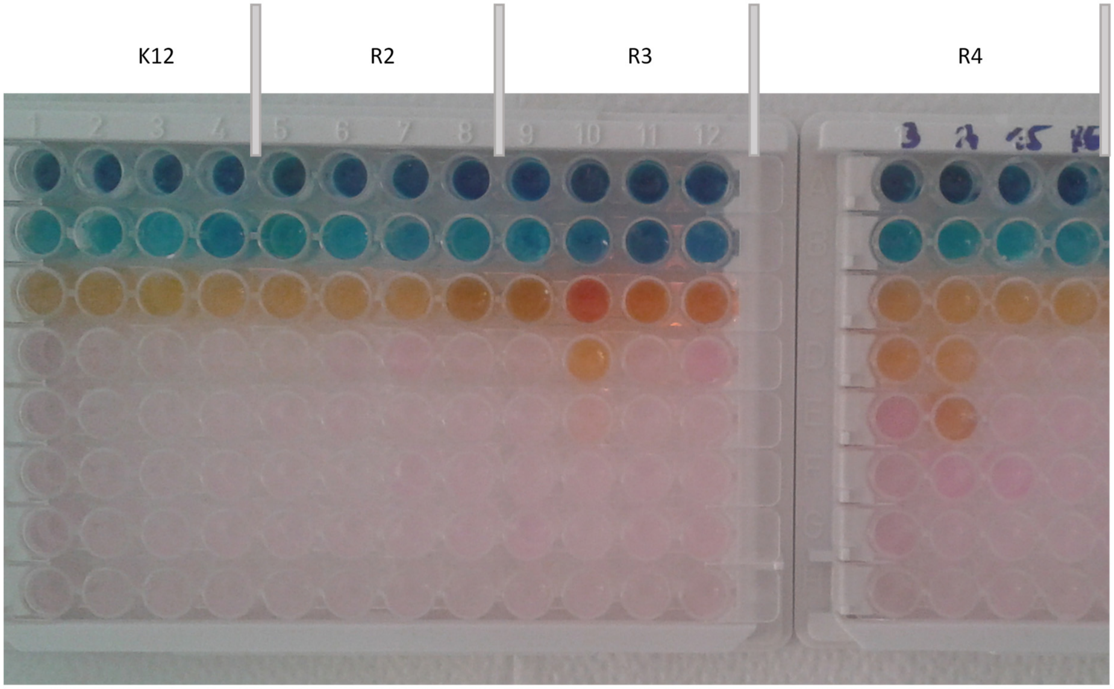

Figure S2. Examples of MIC for investigated K12, R2, R3, R4 strains of *E. coli.* on microplates with different concentration of studied antibiotic kanamycine (well 1, 5, 9, 13), bleomycine (well 2, 6, 10, 14), streptomycine (well 3, 7, 11, 15), and ciprofloxacine (well 4, 8, 12, 14) (mg L^{-1}).

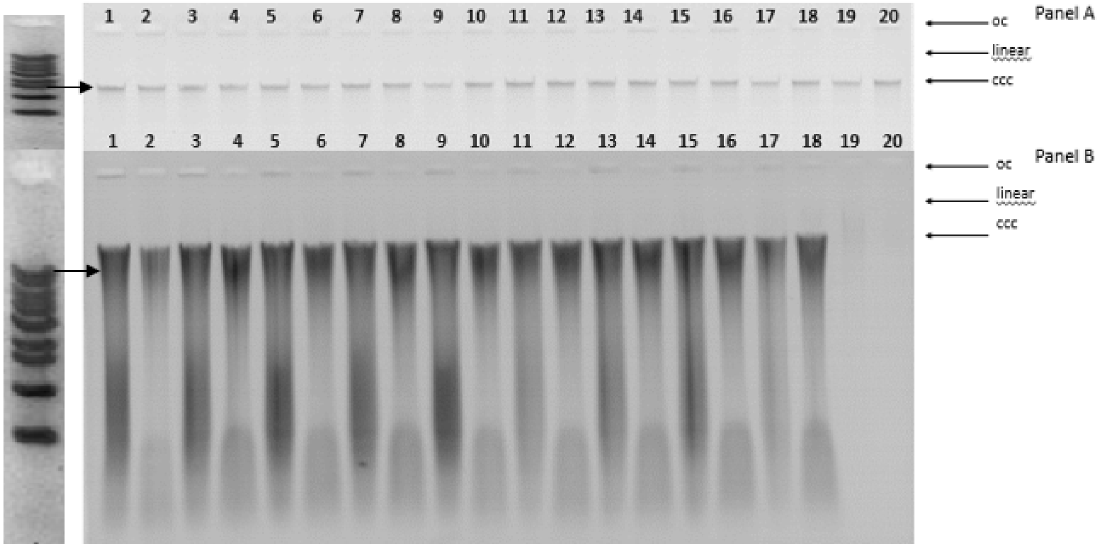

Figure. S3. An example of agarose gel electrophoresis separation of isolated from R4 strains unmodified and modified plasmid with α-amidoamids solution and digested (or not) with repair enzymes Fpg. Lanes: 1–20 (control R4 modified plasmid- (Panel A), modified plasmid (panel B): F- digested with Fpg protein. An additional well contains a Quick-Load® 1 kb Extend DNA Ladder (New England Biolabs, Ipswich, MA, United States).

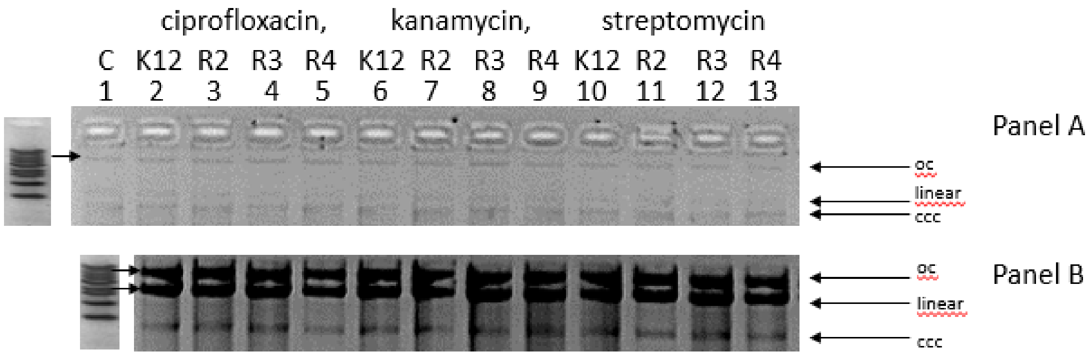

Figure S4. Example of agarose gel electrophoresis separation of plasmids (lanes 2–13) isolated from K12, R2, R3, and R4 strains and reacted with antibiotics: ciprofloxacin, kanamycin, streptomycin, and not digested by repair enzyme Fpg (Panel A) and (panel B): plasmid reacted with antibiotics digested with Fpg protein. Lanes: C –control plasmid (not reacted with antibiotics). An additional well contains a Quick-Load® 1 kb Extend DNA Ladder.

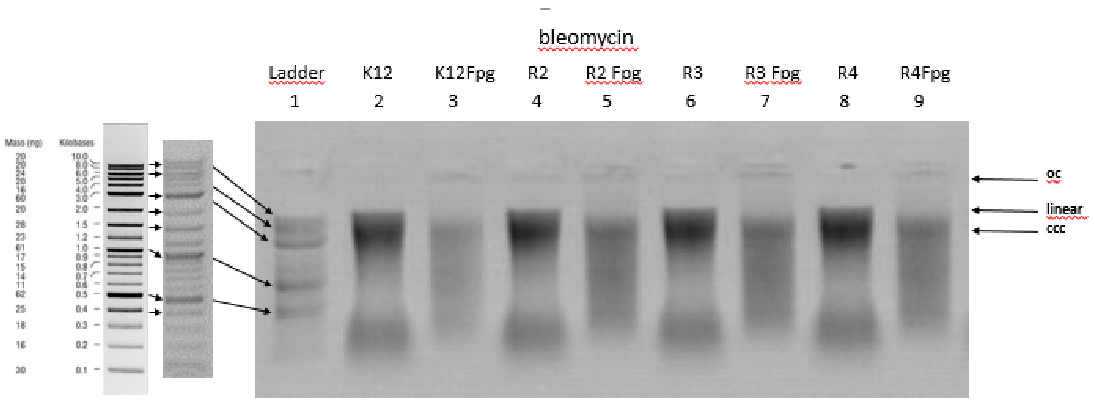

Figure S5. Example of agarose gel electrophoresis separation of plasmids (lanes 2, 4, 6, 8) isolated from K12, R2, R3, and R4 strains and reacted with antibiotic: bleomycin and digested by repair enzyme Fpg (lanes 3,5,7,9) Lanes 1kb-ladder, NEB, Cat. No. (N0559S), (the size of the five mainly bold bands on the DNA ladder from bottom to top are 400, 500, 1000, 1500 and 2000 bp, respectively).

© 2020 by the authors. Submitted for possible open access publication under the terms and conditions of the Creative Commons Attribution (CC BY) license (http://creativecommons.org/licenses/by/4.0/).
